# Morphological diversity of indigenous wild pomegranate (*Punica granatum* L. var. *spinosa*) accessions from northeast of Iran

**DOI:** 10.1002/fsn3.3135

**Published:** 2022-12-07

**Authors:** Seyed Hasan Ashrafi, Hojatollah Bodaghi, Mehdi Rezaei

**Affiliations:** ^1^ Department of Horticulture Science and Plant Protection, College of Agriculture Shahrood University of Technology Shahrood Iran

**Keywords:** breeding, genetic diversity, PCA, wild pomegranate

## Abstract

Wild pomegranate is a valuable edible species in the plant ecosystem of the Hyrcanian forests and the northern plains of the Caspian coast in Iran. The genetic diversity of these wild pomegranates can be effective in pomegranate breeding programs and germplasm conservation. In the present study, morphological diversity in 103 wild pomegranates (*Punica granatum* L*.* var. *spinosa*) in the northeastern area of Iran was studied using 46 traits related to trees, flowers, and fruits. The results showed that the fruit weight ranged from 17.93 to 99.9 g with an average of 48.92 g, the total aril weight ranged from 0.54 g to 64.78 g with an average of 24.25 g, and the weight of 100 arils was between 4.89 and 46.21 with an average of 13.79. The fruit cracking percent, crown shape, aril juiciness, calyx, and corolla colors show a high coefficient of variation (CV > 70%). Based on PCA results, fruit weight and total aril weight, peel weight, and fruit length and diameter were important on determining differences among accessions. In biplot analysis, genotype distribution was determined by two main factors. In cluster analysis, the studied accessions were divided into two different major clusters and two subclusters in each one. The results showed a high diversity of important pomological traits in wild pomegranates such as fruit weight, fruit cracking percent, crown shape, total aril weight, aril juiciness corolla, and calyx color that can be used in breeding programs to improve pomegranate juice quality and marketability.

## Graphical abstract

The aim of this study was to investigate the morphological diversity of 103 indigenous wild pomegranate as one of the most important species in the plant ecosystem of the Hyrcanian forests and the northern plains of the Caspian coast in Iran. Based on the results, fruit weight and total aril weight, peel weight, and fruit length and diameter were important in determining differences among accessions. The results showed high morphological diversity in wild pomegranates that can be used in breeding and conservation programs.

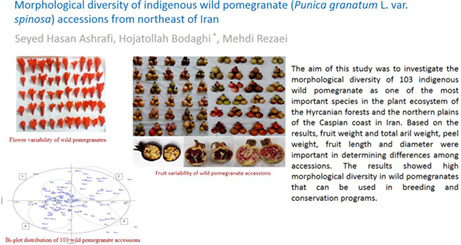

## 1 INTRODUCTION

Pomegranate, *Punica granatum* L., is a small tree that is cultivated well in the arid area of subtropical and Mediterranean climates (Yilmaz et al., 2021). It is one of the famous oldest cultivated fruits in west Asia and the Middle East. There are several varieties of *P. granatum* L. that are used for nutrition and medicinal properties and ornamental value (Aziz et al., 2020). Wild pomegranates, *P. granatum* L. var. *spinosa*, have much smaller arils and fruit, thicker rinds, and higher acidity than cultivated ones (Kher, 1999; Sharma & Sharma, 1990). These wild pomegranates have a lot of suckers, irregularly thorny twigs, and small leaves (Singh & Gupta, 2018; Thakur et al., 2011). The harvested fruits from these trees are used by local people to prepare various pomegranate pastes. These pastes are an essential part of the local cuisine, such as vegetable processing, sour chicken, Fosanjan, and conserved olives, and they are also considered tasting kebab (Mirjalili & Poorazizi, 2015). In addition, these fruits are noted as a great source of natural antioxidants and health‐promoting constituents like anthocyanins, phenolics, and flavonoids (Thakur et al., 2020) and they have some antitumoral effects (Sineh Sepehr et al., 2012).

The most critical step in any breeding program is screening and identifying superior genotypes, which is very costly and time‐consuming. Diversity is the basis of all selection and crop improvement requires genetic diversity. As the diversity increases, the selection scope also extends. It is better to identify and screen superior genotypes in the same climatic region as a collection. However, wild genotypes do not have acceptable yields, so they have intrigue breeders. Still, they are valuable in some quantitative and quality traits such as resistance to abiotic and biotic stresses and nutritional quality (Zuriaga et al., 2022). Wild pomegranates have broad geographical distributions from Iran and Turkmenistan to northern India (Chandra et al., 2010). In Iran, wild pomegranates are adapted very well to various environmental conditions, and they are dispersed around all of the plains in coast Caspian and Hyrcanian forests (Khadivi et al., 2020). Today, with human exploitation in Iran, the natural resources of wild pomegranate populations are often endangered (Khan et al., 2014). Hence, studies on the wild pomegranate diversity are valuable to find superior genotypes for breeding programs and to establishing core collections for germplasm conservations and more detailed studies (Arlotta et al., 2022).

Numerous studies have been conducted on the morphological diversity of fruits and the biochemical composition of domestic and wild pomegranates in different parts of the world. For example, in a study on morphological variability of wild pomegranate in northern parts of Iran, Khadivi et al. (2020) reported a high diversity in fruit weight, fruit peel color, aril color, fresh arils weight, and total soluble solids. A wide variation in fruit size, fruit peel, anthocyanin content, total soluble solids, aril juice content, and seed hardness was reported in cultivated pomegranates in Iran (Sarkhosh et al., 2009). Similar phenotypic variation has been reported within wild pomegranate collections in Pakistan (Aziz et al., 2020; Nafees et al., 2015) and India (Mishra et al., 2016; Singh & Gupta, 2018). A developing of SSRs was introduced as a rapid tool for the identification of pomegranate cultivars in the study of nutraceutical and genetic diversity of novel pomegranate genotypes in comparison to leading commercial pomegranate varieties (Arlotta et al., 2022). In addition, the genetic variability in pomegranate cultivars was reported using both nuclear (SSRs) and chloroplast genetic regions (*trnH‐psbA* spacer, and *matk* gene; Shahsavari et al., 2022). However, diversity in flower characteristics has received less attention.

The morphological diversity of wild pomegranates from the northeast region of Iran is lacking. The objective of the current study was to determine the morphological variability of trees, flowers, and fruits among wild pomegranate accessions in the northeast region of Iran to inform best strategies for incorporating beneficial traits into pomegranate breeding programs as well as aid germplasm conservation efforts.

## 2 MATERIALS AND METHODS

### 2.1 Plant materials

**FIGURE 1:**
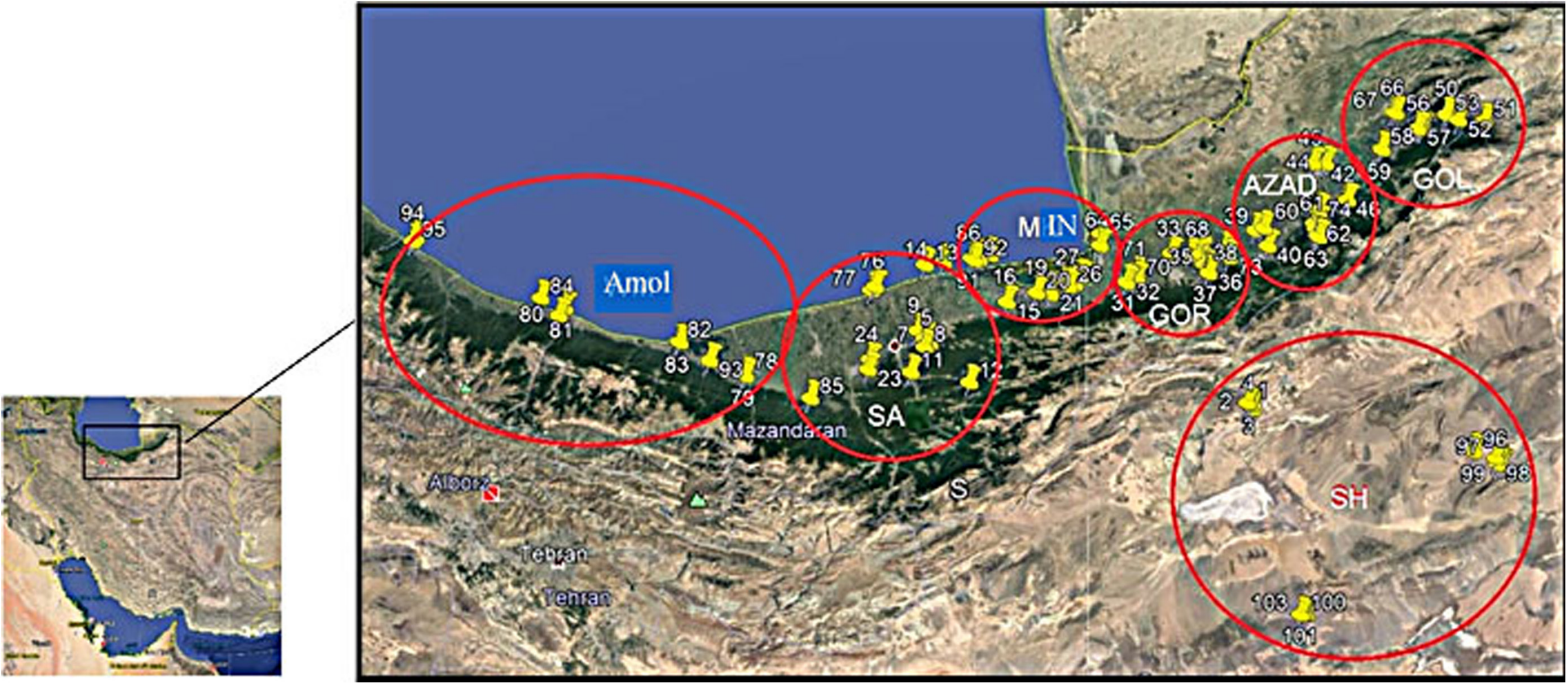
Geographical map of sampling locations of 103 wild pomegranate accessions in Iran.

To study the phenotypic variability of wild pomegranate in the northeastern area of Iran, 103 wild pomegranate accessions (*P. granatum* var *spinosa*) from natural habitats of Semnan, Mazandaran, and Golestan provinces were assessed using pomological and morphological traits. Geographical coordinates and altitude corresponding to each surveyed area are presented in Figure 1 and Table 1. To avoid the possibility of sampling and collecting clones of the selected accessions, at least a 150 m distance was considered between the accessions in each collection site.

**TABLE 1:** Accessions numbers, area, code, and sampling altitudes of 103 wild pomegranates in Golestan, Mazandaran, and Semnan provinces in Iran.

No.	Province	Area	Code	Accession number	Altitude (m)
1	Golestan	Azadshar	Azad	17	29 to 425
2	Golestan	Golestan forest	GOL	12	294 to 884
3	Golestan	Gorgan	Gor	22	−24 to 160
4	Mazandaran	Amol, Ramsar, Nour	Amol	11	−6 to 332
5	Mazandaran	Miankaleh	MIN	15	−24 to 28
6	Mazandaran	Sari	SA	14	−25 to 375
7	Semnan	Shahrood	SH	12	771 to 1258

### 2.2 Measured traits

**FIGURE 2:**
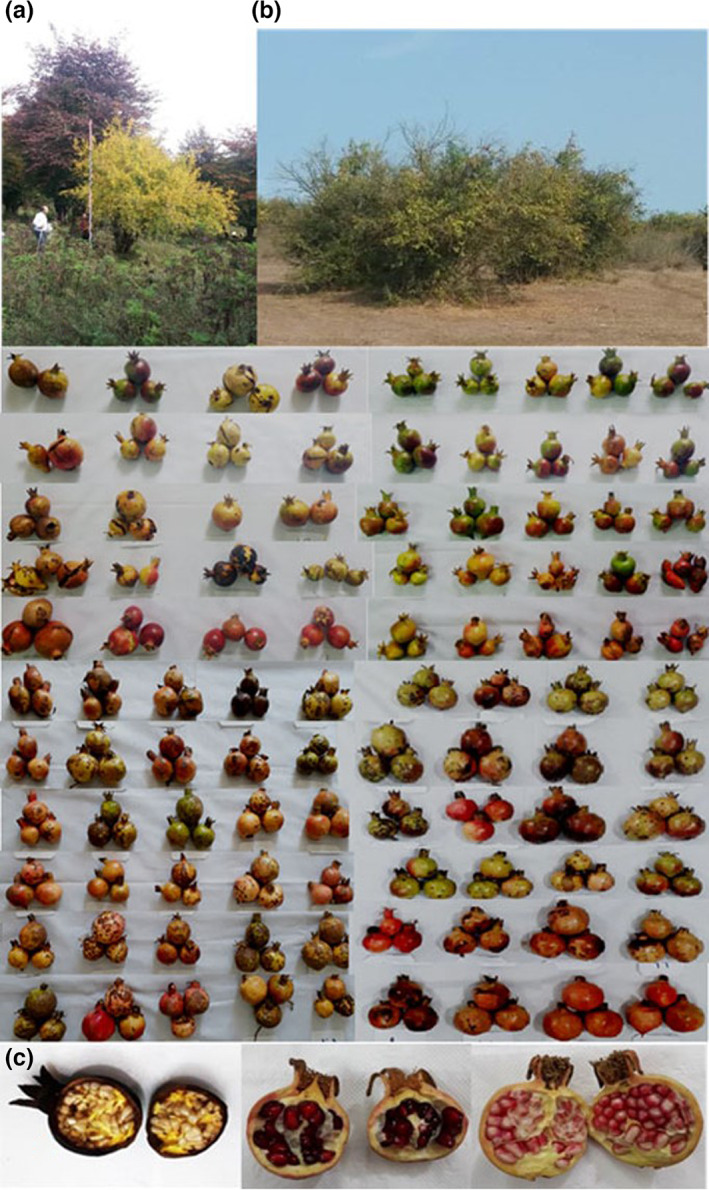
The pictures of tree growth habit and fruit variability of wild pomegranate accessions from the northeast area of Iran. (a) Wild pomegranate tree height, (b) Tree growing in natural habitat, and (c) Wild pomegranate fruits.

Forty‐six morphological characteristics related to trees, leaves, thorns, flowers, and fruits were evaluated based on the pomegranate descriptor (UPOV, 2012). At first, the geographical coordinates and altitude of each wild pomegranate accession were recorded with a GPS device, and coding was done for each tree (Figure 1). Tree height was measured with an index (Figure 2a), and the characteristics related to tree vigor, growing habits, type of flowers, and thorns were evaluated based on the respective descriptor. In total, 25 full‐maturity fruits, 25 leaves, and 25 flowers were randomly sampled to assess each accession. The traits, including fruit weight, aril weight, and peel weight, were measured using an electronic laboratory balance with 0.01 g precision (Bonvoisin, US), and fruit crown length and diameter, fruit crown width, aril length and diameter, peel thickness, leaf length and width, petiole length, and fruit length and diameter were measured using a digital caliper (Insize Co.). Flower‐related traits were recorded in the first series of bell‐shaped flowers (fertile flowers) in late spring. Fruit‐related traits were assessed in fully ripened fruit in autumn.

### 2.3 Statistical analysis

Descriptive analysis of each trait, including minimum, maximum, mean, variance, and coefficient of variation (CV%: SD/mean*100), was analyzed by SPSS software version 22 (SPSS Inc., Chicago, II., US). Relationships among the accessions were investigated using principal component analysis (PCA) using PAST software. Cluster analysis was also done by PAST software with the Ward method. The calculation of distances was performed after Z‐score standardization. A scatter plot among populations was created according to the first two principal components (PC1 and PC2) using PAST software. The heatmap clustering was drawn by “heatmap” packages in Rstadio ver.1.0.136 software.

## 3 RESULTS AND DISCUSSION

### 3.1 Morphological traits description

A description of the analysis of morphological traits of 103 wild pomegranate accessions was presented in Table 2. The results showed significant differences among the studied accessions based on all the characters recorded (*p* < .01). The high coefficient of variance (CVs) of a trait indicates excessive diversity in the population, indicating its higher opportunity in selection at a breeding program. The range of CVs was from 9.04 (in calyx length‐to‐width ratio) to 292% (in cracking percent), with an average of 35.97% (Table 2). Among the studied traits, in order, fruit cracking percent, crown shape, corolla color, calyx color, aril juiciness, aril color, length of thorn, weight of arils in a fruit, weight of 100 arils, flower shape, carob moth, aril hardiness, fruit over color, and fruit weight have the high CVs (more than 39%).

**TABLE 2:** Descriptive statistics for the morphological traits utilized in the studied wild pomegranate accessions

Num.		Traits	Unit	Abbr.	Min	Max	Mean	Var.	CV%
1	Tree	Height	m	TH	2.5	6.5	4.29	0.88	21.89
2		Vigor	Code	V	5	7	6.41	0.83	14.22
3		Growing habit	Code	GH	1	3	2.61	0.63	30.44
4	Leaf	Leaf length	mm	LL	27.17	67.18	50.07	73.84	17.16
5		Leaf width	mm	Lwi	9.86	21.01	14.37	4.12	14.13
6		Petiole: Length	mm	PL	1.56	8.46	4.61	1.24	24.20
7		Petiole: Color	Code	Pco	3	5	4.61	0.63	17.23
8		Leaf shape	Code	LSh	3	5	4.76	0.41	13.52
9		Tip leaf shape	Code	TLsh	1	2	1.30	0.21	35.42
10		Leaf number per node		LN	6	10	6.89	0.93	14.06
11	Thorn	Big thorn length	mm	BThL	0	32.16	11.59	39.59	54.29
12		Small thorn length	mm	SThL	2.8	12.8	4.48	1.99	31.49
13	Fruit	Fruit weight	g	FW	17.93	99.90	48.92	366.4	39.12
14		Fruit length	mm	FL	30.40	60.93	42.33	36.38	14.24
15		Fruit width	mm	Fwi	25.32	58.10	43.28	42.06	14.98
16		Fruit: length/width ratio		RLWi	0.81	1.49	0.98	0.01	9.89
17		Fruit: length of crown	mm	Lcr	7.01	23.08	15.21	7.92	18.50
18		Fruit: width of crown	mm	Wcr	5.37	28.30	17.09	23.77	28.53
19		Crown diameter	mm	CrD	6.97	18.35	13.76	4.36	15.16
20		Fruit over color	Code	Fco	1	7	3.47	2.38	44.47
21		Crown shape	Code	CrSh	1	5	2.08	2.96	82.46
22		Peel weight	g	PW	7.75	47.37	23.50	63.70	33.96
23		Thickness of peel	mm	TS	1.41	5.44	3.16	0.65	25.66
24		Cracking percent	%	CP	0	91.7	6.44	354.80	292.48
25	Aril	Fruit aril weight	g	AW	0.54	64.78	24.25	146.84	49.95
26		Weight of 100 aril	g	WA100	4.89	46.21	13.79	44.86	48.55
27		Aril length	mm	AL	6.04	10.69	8.16	0.99	12.20
28		Aril diameter	mm	AD	2.59	8.68	5.19	1.05	19.72
29		Aril juicy	Code	AJ	1	7	2.84	4.25	72.53
30		Aril hardiness	Code	AH	1	5	3.19	1.94	43.59
31		Aril color	Code	Aco	0	5	3.15	3.30	57.65
32		Aril percent	%	RAP	2.41	68.09	47.81	82.09	18.94
33		Aril length/width Ratio		ALWi	1.11	2.83	1.60	0.060	15.26
34	Flower	Flower size	Code	Fsi	3	5	4.00	1.01	25.06
35		Flower shape	Code	FSh	1	5	3.73	3.37	49.12
36		Flower type	Code	FT	1	9	8.14	6.16	30.48
37	Percent of bell‐shaped flower		%	PBF	20	45	35.87	25.20	13.99
38		Calyx length	mm	CL	29.22	46.36	38.86	13.31	9.38
39		Calyx width	mm	Cwi	10.12	15.82	12.20	1.93	11.39
40		Calyx length/width ratio		CLWi	2.32	3.86	3.20	0.08	9.04
41		Calyx color	Code	Cco	1	5	2.08	2.57	76.81
42		Corolla color	Code	CoCo	1	5	2.04	2.65	79.53
43		Petal length	mm	PL	14.77	26.86	22.26	10.72	14.70
44		Petal width	mm	Pwi	9.81	22.22	15.89	8.40	18.24
45		Petal shape	Code	PSh	3	7	5.54	1.66	23.25
46		Carob moth	Code	CM	0	1	0.81	0.15	47.79

The fruit weight with means of 48.92 g ranged from 17.93 g (SA8) to 99.90 g (SH11). The fruit weight in Iranian cultivated pomegranates is 103.28–407.59 g (Khadivi & Arab, 2021), which indicates that wild pomegranate accessions predominantly are smaller in size than commercial varieties (Figure 2). The fruit weight in wild pomegranates from India has been reported in the range 80.50–85.17 g (Kher, 1999), and 55.10–83.50 g with averages of 64 g (Thakur et al., 2011). Khadivi et al. (2020) reported that the fruit weight of wild pomegranates in Iran ranged from 19.20 to 185.00 g with an average of 59.89 g. Genetic variation and pedoclimatic conditions can influence fruit weight (Martinez et al., 2006). In this study, since all the genotypes were evaluated in the different geographical zone, variation in fruit weight can be influenced by genetic and climatic conditions.

The weight of arils in a fruit ranges from 0.54 (AZAD2) to 64.87 g (SH11) with a mean of 24.25 g. The weight of 100 arils ranged from 4.89 to 46.21 g (MIN3), with a mean of 13.79 g. It is a standard for determining the size of the aril so that the heavier the weight of 100 arils, the larger the arils, and vice versa. The total weight of pomegranate aril is considered one of the most important economic criteria for the industrial production of pomegranates (Maestre et al., 2000).

The fruit length with an average of 42.33 mm ranged from 30.40 mm (SH96) to 60.93 mm (SA11). Also, the fruit width with an average of 43.28 mm varied from 25.32 mm (AZAD4) to 58.10 mm (MIN11). These properties are considered in the grading and marketability of pomegranate fruit. In addition, these valuable data are used to design and selection of proper packaging during storage and handling (Valero & Ruiz‐Altisent, 2000). Another important trait in pomegranate fruit is the peel thickness, which in the studied accession with a CV of 25.66% and an average of 3.16 mm showed a range between 1.41 mm (GOL3) and 5.44 mm (AZAD5). The fruits with thick peel are intended for extended shelf life and longevity (Khadivi‐Khub, 2015). Furthermore, fruits with thick peels might have the ability to resist peel cracking. A thick peel enclosing the edible arils protects the fruits against pests and pathogens that contaminate the fruits via these cracks (Jalikop et al., 2006), while pomegranate with thin peels is considered more for processing (Khadivi et al., 2018).

**FIGURE 3:**
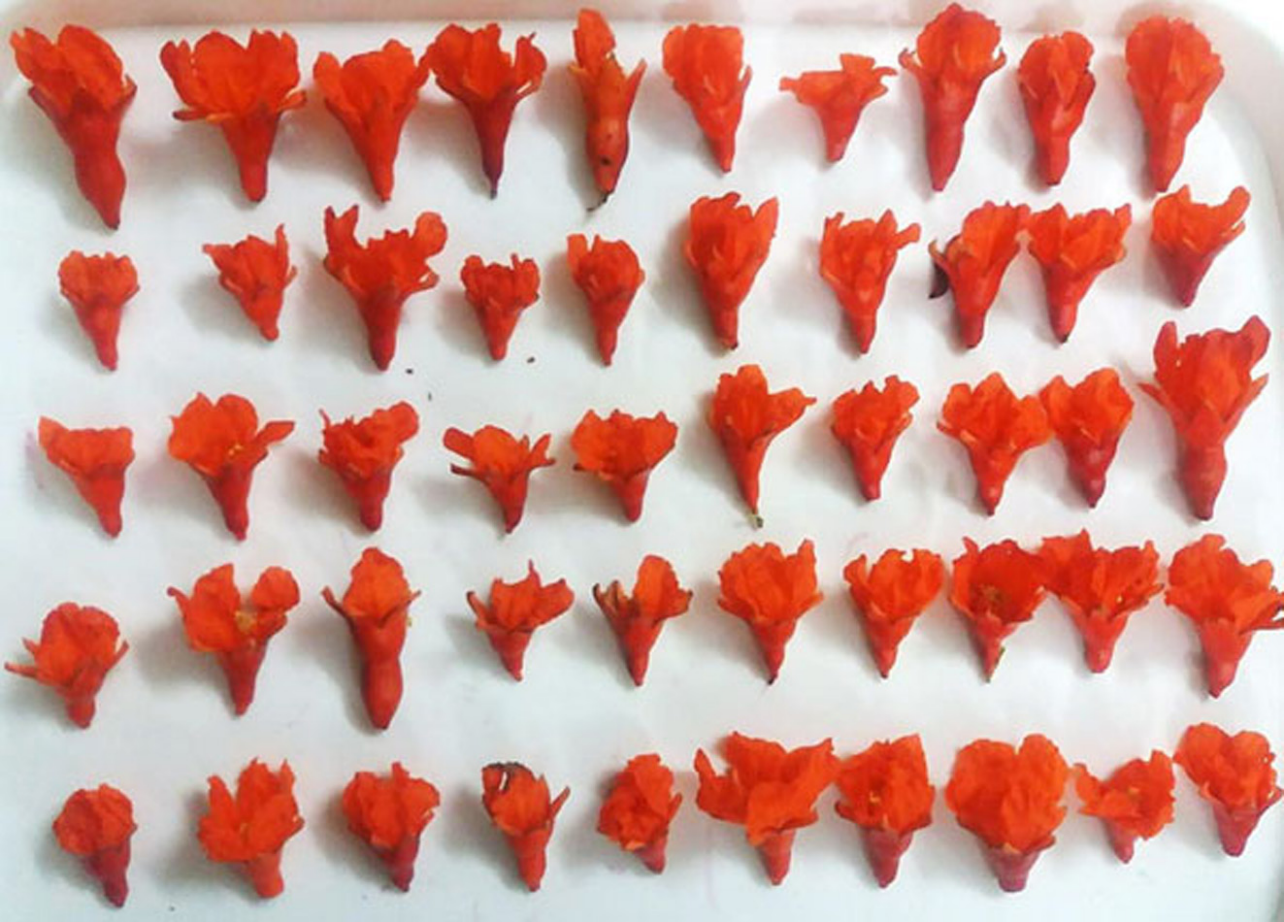
The pictures of flower variability of wild pomegranate accessions from the northeast area of Iran.

Tree height with a mean of 4.29 m varied from 2.5 to 6.5 m. Leaf length, with an average of 50.07 mm, ranged from 27.18 to 67.19 mm. The leaf width varied from 9.86 to 21.01 mm. The petiole length, with an average of 4.61 mm, ranged from 1.57 to 8.46 mm. The percentage of fertile flowers was between 20 and 45% with an average of 35.87%. Trees predominantly have spreading growth habits (84) and strong growth vigor (73) (Table 3). Most accessions have oblong leaves (88) and strong acute tip leaf shape (70) with red petioles (81). The fruit over color showed high variability and included reddish green (18), yellow (30), greenish yellow (8), reddish‐yellow (30), red (6), and black (1) (Figure 2). Three forms of fruit crown shape were observed, including loose (73), semi loose (6), and closed (24). Eighty‐five pomegranate accessions showed no signs of carob moth larva activity in fruits. Over 47 of accessions have very low aril juice, which reduces their use in local cuisine. Also, 54 of the arils in accessions were semihard. The predominance of flowering in pomegranate accessions occurs in the terminal spur (93). The flower size of 50 accessions was medium and 53 accessions was large, and 67 of the accessions had cylinder flowers (Table 3 and Figure 3). The petal shape of 51 pomegranate accessions was oblong. The color of calyx and corolla in 66 and 73 of them were orange, respectively.

**TABLE 3:** Frequency distribution of qualitative traits in wild pomegranate accessions from the northeast area of Iran

Traits	0	1	2	3	4	5	6	7	9
Tree vigor		–	–	Weak (0)	–	Medium (30)	–	Strong (73)	
Growing habit		Upright (19)	–	Spearing (84)	–	Weeping (0)	–	–	
Leaf shape		Ovate (0)	–	Oblate (15)	–	Oblong (88)	–	–	
Tip leaf shape		Strong Acute (70)	Moderate Acute (33)	Right Angle (0)	Moderate Obtuse (0)	Strong Obtuse (0)	–	–	
Petiole: Color		Light Green (0)	Green (10)	Light Red (12)	–	Red (81)	–	–	
Fruit over color		Green (10)	Reddish Green (18)	Yellow (30)	Greenish Yellow (8)	Reddish Yellow (30)	Red (6)	Black (1)	
Fruit crown shape		Loose (73)	–	Semiloose (6)	–	Closed (24)	–	–	
Carob moth	Positive (18)	Negative (85)	–		–	–	–	–	
Aril juicy		Very Low (47)	–	Low (26)	–	Medium (19)	–	High (11)	
Aril hardiness		Soft (20)	–	Semihard (54)	–	Hard (29)	–	–	
Flower type		Lateral Spur (10)	–	–	–	–	–		Terminal Spur (93)
Flower size		Small (0)	–	Medium (50)	–	Large (53)	–	–	
Flower shape		Open Vase (30)	–	Oblong (6)	–	Cylinder (67)	–	–	
Petal shape		–	–	Circular (11)	–	Oblong (51)	–	Long Oblong (41)	
Calyx color		Orange (66)	–	Red Orange (16)	–	Red (21)	–	–	
Corolla: color		Orange (73)	–	Red Orange (12)	–	Red (18)	–	–	

### 3.2 Principal component analysis (PCA)

Principal component analysis is a technique often used to facilitate data exploration and visualization with emphasis on variation and showing strong patterns in the dataset. PCA simplifies the complexity of high‐dimensional data by maintaining trends and patterns. It does this by converting data into fewer dimensions, which acts as a summary of features. High‐dimensional data are prevalent in biology and appear with several features. PCA reduces data with its geometric layout in lower dimensions called principal components (PCs), intending to find the best summary of data using the finite number of PCs. The first PC is selected to minimize the total distance between the dataset.

**FIGURE 4:**
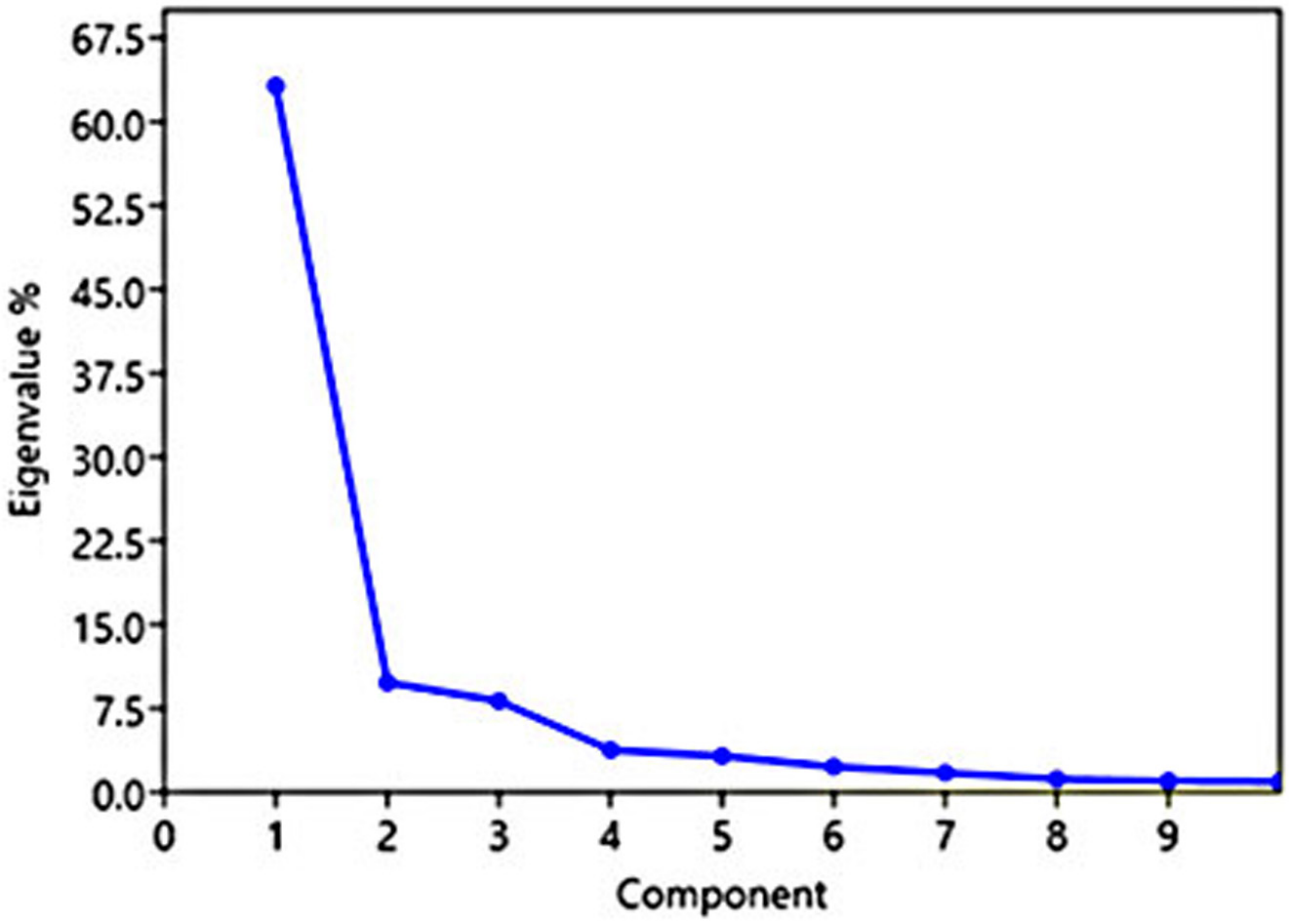
The eigenvalue of first 10 principal components (PCs) of 46 morphological traits in 103 wild pomegranate accessions.

In this study, 95.39% of the variance explains by the initial 10 PCs. Khadivi et al. (2020) reported 72.06% of the total variance in 23 PCs in wild pomegranate accessions of the northern parts of Iran. In the PC1, seven traits, including fruit weight (0.74), fruit aril weight (0.45), peel weight (0.26), fruit width (0.24), fruit length (0.20), and weight of 100 arils (0.12), whose eigenvalues were more than 0.1, could justify 63% of the total variance (Figure 4 and Table 4). These features were the most effective traits for isolating and identifying the studied accessions. Furthermore, these traits are economically important and can be used as a valuable tool for selecting new genotypes or cultivars with high performance. Zarei et al. (2018) reported the main traits in the first PCs, including arils weight, fruit weight, peel weight, number of arils, aril length, aril width, aril thickness, aril length, aril width, fruit length, and fruit width in the commercial cultivars of pomegranate. In the PC2, there are six effective traits, including leaf length (0.67), percentage of bell‐shaped flowers (0.27), fruit peel weight (0.28), and flowering type (0.10), with a positive coefficient, and aril weight (−0.25) and aril percentage (−0.50) with a negative coefficient, which justifies 9.82% of the variance. In the PC3, there are 10 effective traits, including the percentage of bell‐shaped flowers, leaf length, thorn length, total aril weight, and aril percentage with a positive coefficient, and fruit weight, fruit length, fruit crown width, and fruit peel weight with a negative factor coefficient that explained 8.16% of the variance (Table 4). These data confirmed that fruit‐related characteristics play an essential role in determining genotypic differences and selecting superior plant material in the breeding programs (Khadivi et al., 2020; Khadivi & Arab, 2021; Zamani et al., 2013).

**TABLE 4:** The eigenvalue of each trait in three principal components (PCs)

	PC_1_	PC_2_	PC_3_		PC_1_	PC_2_	PC_3_
TH	0.007	0.035	0.023	PW	0.261^a^	0.283^a^	−0.245^a^
V	−0.001	0.027	0.041	TS	0.000	0.028	−0.035
GH	−0.001	0.017	0.041	AW	0.453^a^	−0.257^a^	0.141
FT	−0.017	0.101^a^	0.031	WA100	0.127^a^	−0.074	−0.306^a^
PBF	0.013	0.272^a^	0.204^a^	AL	0.015	−0.016	−0.022
LL	0.082	0.674^a^	0.434^a^	AD	0.007	−0.015	−0.051
LWI	−0.004	0.031	0.003	AJ	0.020	−0.062	−0.034
PL	0.001	0.002	−0.045	AH	−0.014	0.016	0.018
PCO	0.000	0.030	0.022	Aco	0.004	0.023	−0.057
LSH	−0.005	0.030	0.023	RAP	0.174	−0.503^a^	0.621^a^
TLSH	0.001	−0.016	−0.017	ALWi	0.000	0.001	0.010
LN	0.008	0.025	−0.004	Fsi	0.014	0.009	−0.008
BTHL	0.089	0.097	0.279^a^	FSh	0.030	0.054	0.044
FW	0.744^a^	0.077	−0.137	CL	0.066	0.019	0.056
FL	0.201^a^	−0.004	−0.207^a^	Cwi	0.019	−0.001	0.031
FWI	0.241^a^	0.039	−0.021	CLWi	0.001	0.002	−0.004
RLWI	−0.001	−0.001	−0.005	Cco	−0.020	−0.052	−0.081
LCR	−0.021	0.066	−0.022	CoCo	−0.021	−0.057	−0.072
WCR	−0.047	−0.089	−0.119	PL	−0.001	0.058	−0.014
CRD	0.007	0.004	−0.093	Pwi	−0.035	0.002	−0.073
FCO	−0.011	−0.013	−0.053	PSh	0.024	0.049	0.050
CRSH	0.003	−0.008	0.033	CM	0.000	−0.011	0.000

^a^ Values above 0.1 were considered significant.

**FIGURE 5:**
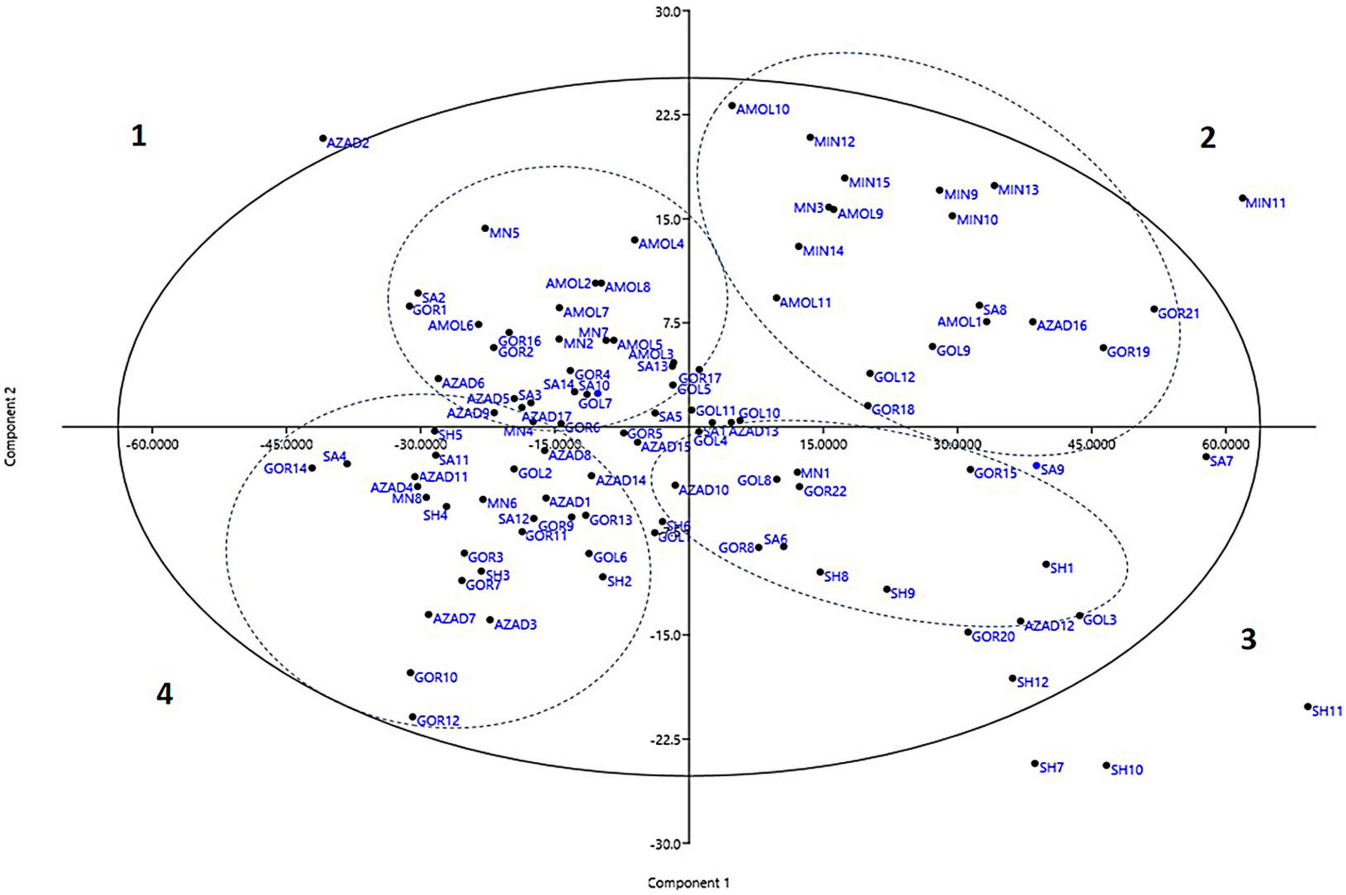
Biplot distribution of 103 wild pomegranate accessions based on two main principal components.

Biplot analysis represented a two‐dimensional distribution of studied accessions based on effective traits in the first and second PCs. The closeness of genotypes in one area of the biplot indicates their genetic similarity. Biplot analysis in wild pomegranate accessions was performed based on two main principal components with 63.23% and 9.82% of the variance, respectively (Figure 4). Pomegranate accessions were well distributed on four sides of the biplot, which indicates the high genetic diversity of studied wild pomegranates. In PC1, by starting from a negative toward a positive value, the accessions show a gradual increase in fruit weight, aril weight, and fruit length and width. In addition, by starting from negative values toward positive ones, in PC2, the accessions have a gradual increase in leaf length, fruit peel weight, and their arils percent, and arils weight decreased (Figure 5).

The AZAD2 with the lowest aril weight on the first plot side, MIN11 with high fruit weight (98.24 g) on the second plot side, and SH7, SH10, and SH11 with large fruits and highest total aril weight on the third plot side were the further away from other accessions. The studied wild pomegranate accessions were separable in four biplot regions (Figure 5). In region 1, there are accessions from Amol (2–8), Sari (2, 3, 5, 10, 13, and 14), Gor (1, 2, 4, 6, and 16), MIN (2, 4, 5, and 7), Azad (5, 6, 9, and 17), and Gol (5 and 7). These accessions have similarities in the length and width of the fruit crown and flowering type. The accessions of Amol were dominant in this region. Accessions from MIN (3, 9, 12–15), Amol (1 and 9–11), Gor (17, 18, 19, and 21), Gol (9 and 10–12), Sari (1 and 8), and Azad (13 and 16) were located in the second biplot area. These accessions were more similar in fruit weight, fruit peel weight, thorn length, leaf length, and leaf length. In the third area, there are accessions from SH (1, 8, 9, and 12), Sari (1, 6, 7, and 9), Gor (8, 15, 20, and 22), Gol (3, 4, and 8), and MIN (1). The fruits of these accessions were longer and they had larger arils than other wild pomegranates. In the fourth biplot area, there are accessions from Gor (3, 5, 7, 9, and 11–14), Azad (1, 3, 4, 10, 11, 14, and 15), SH (2–6), SA (4, 11, and 13), MIN (4, 6, and 8), and Gol (2, 6, and 9). The predominant accessions in this group were from the Gorgan region, and they are similar in fruit crown length and width and petal length and width. The information related to samples and variables of a data matrix is displayed geographically in a biplot. Variables are indicated as vectors, linear axes, or nonlinear trajectories, while samples are shown as points. These points may be used to present the level of a categorical variable (Khadivi et al., 2020).

### 3.3 Cluster analysis

**FIGURE 6:**
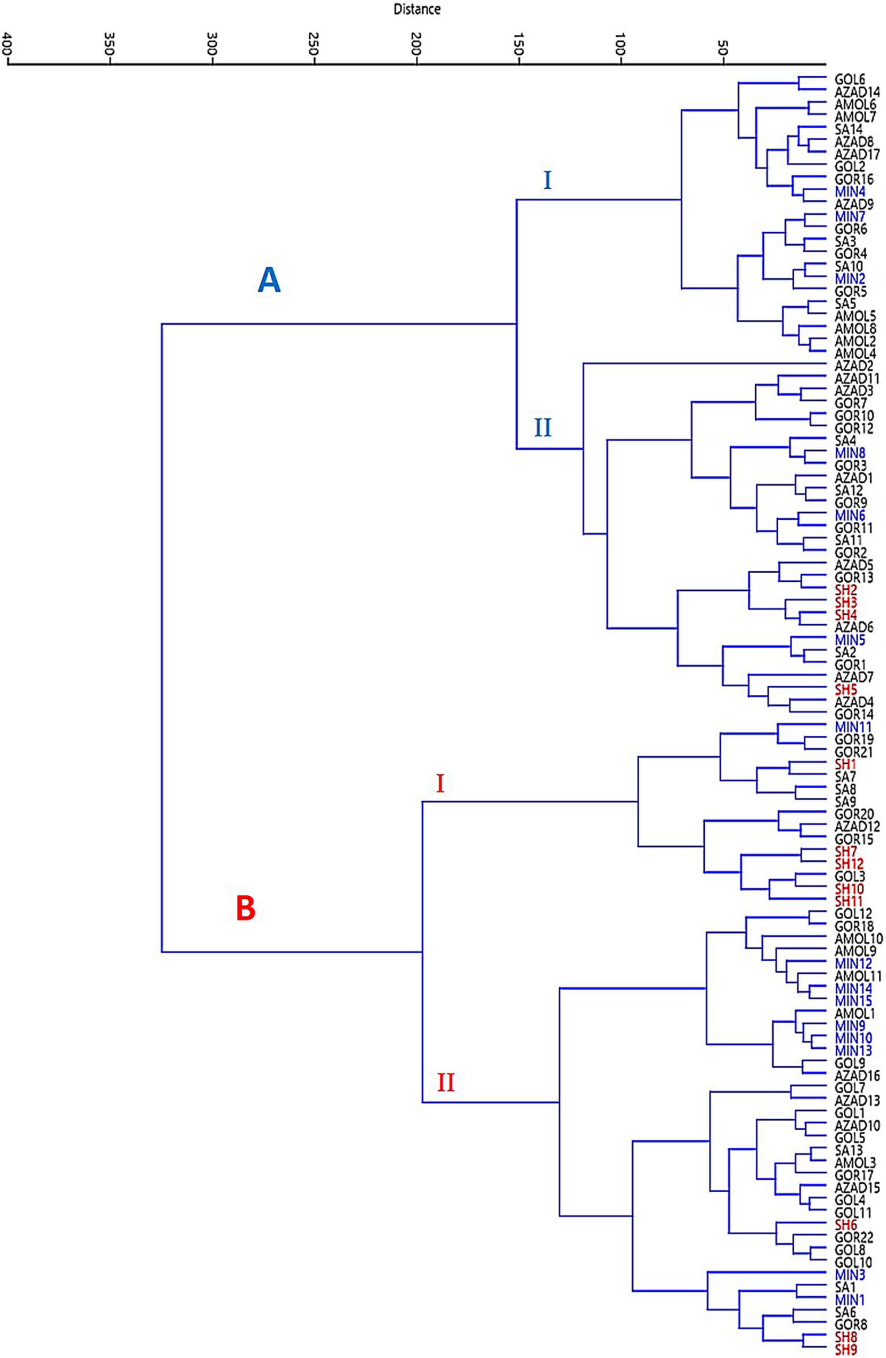
Cluster analysis by 103 wild pomegranate accessions in the northeastern regions of Iran using 45 morphological traits.

Cluster analysis is a technique to group a set of observations in such a way that individuals in the same cluster are more similar to each other than to those in different clusters. Cluster analysis tried to identify similar groups. Unlike PCA, this analysis uses all traits with the same weight. The cluster analysis based on the ward method showed two different significant groups in 103 accessions (A and B) (Figure 6). The first group A was divided into two subgroups I and II, by decreasing the distance from the scale of 150, and the second group B, on the scale of 200, was also divided into two subgroups, I and II. Group A of cluster analysis from Gol6 to Gor14 includes 52 accessions and the second group (B) from MIN11 to SH9 has 51 accessions. Twenty‐three accessions from different studied regions except for Shahrood, with the predominance of accessions of the Amol region, were placed in subgroup A‐I. Subgroup A‐II, from Azad 2 to Gor14, consists of 29 accessions. B‐I subgroup in cluster analysis includes 15 accessions from MIN 11 to SH11. Common features of them include leaf length, peel weight, bell flowering percent, and aril weight. Subgroup B‐II in cluster analysis from Gol12 to SH 9 consists of 36 accessions. In this group, accessions have similar fruit weights, total weight of arils, peel weight, fruit diameter, aril percentage, the weight of 100 arils, and fruit length. The cluster analysis groups are largely confirmed with PCA results.

### 3.4 Scatter plot of populations

**FIGURE 7:**
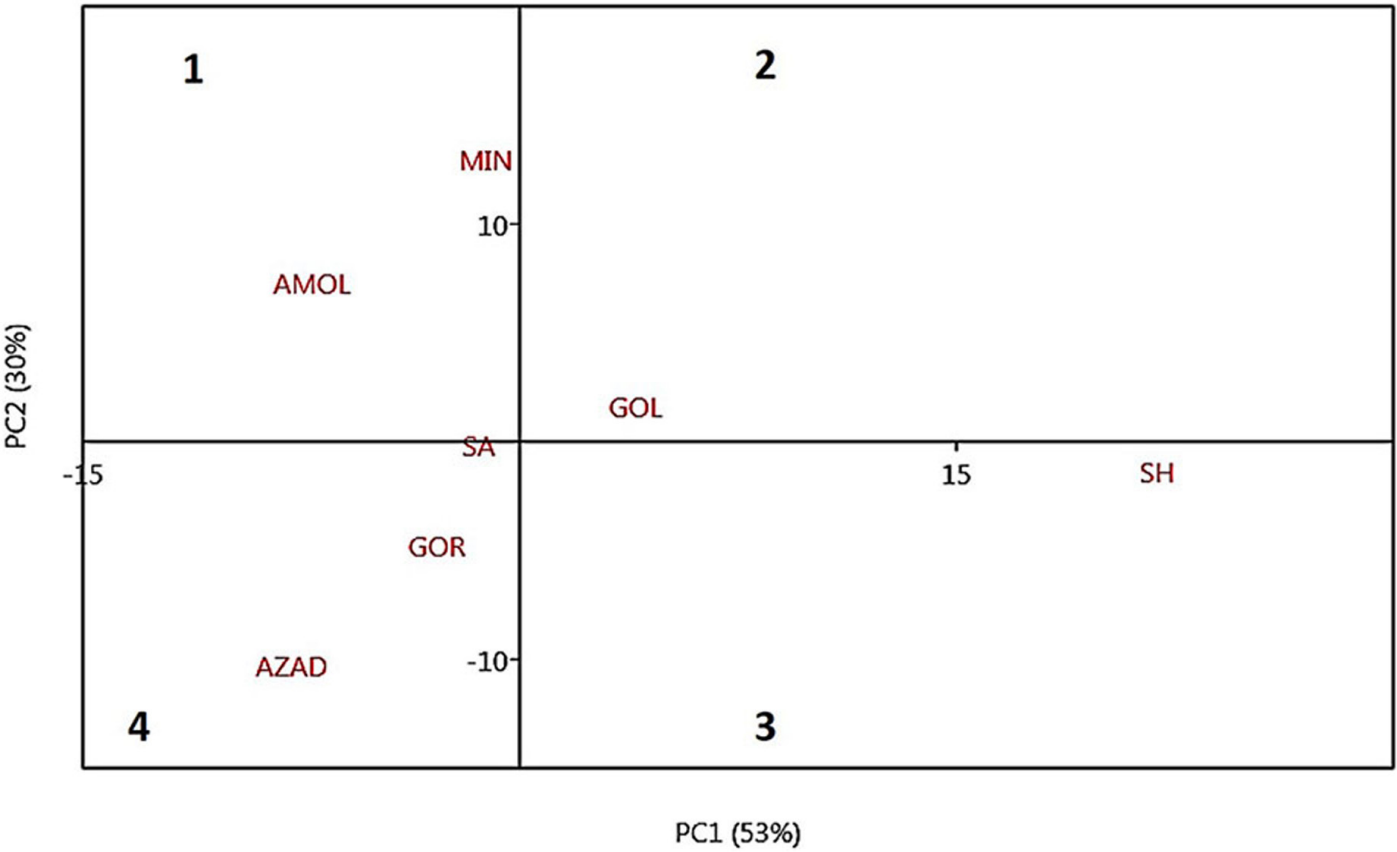
Scatter plot of seven wild pomegranate populations in the northeastern regions of Iran.

Scatter plot analysis was performed to show the distribution of populations based on two main principal components (Figure 7). The vicinity of populations in the plot indicates their genetic similarity, and the high distance indicates high morphological diversity. As seen in the scatter plot, the Amol population was located next to the Miankaleh population in zone 1. Sari, Gorgan, and Azadshahr were located next to each other in the fourth scatter plot area. The Golestan population was in zone 2 of the scatter plot with a short distance from the Sari population. Shahroud population was located in zone 3, far from other populations. Shahroud region, with high altitude, is geographically separated from other studied areas (Figure 1).

### 3.5 Heatmap analysis

**FIGURE 8:**
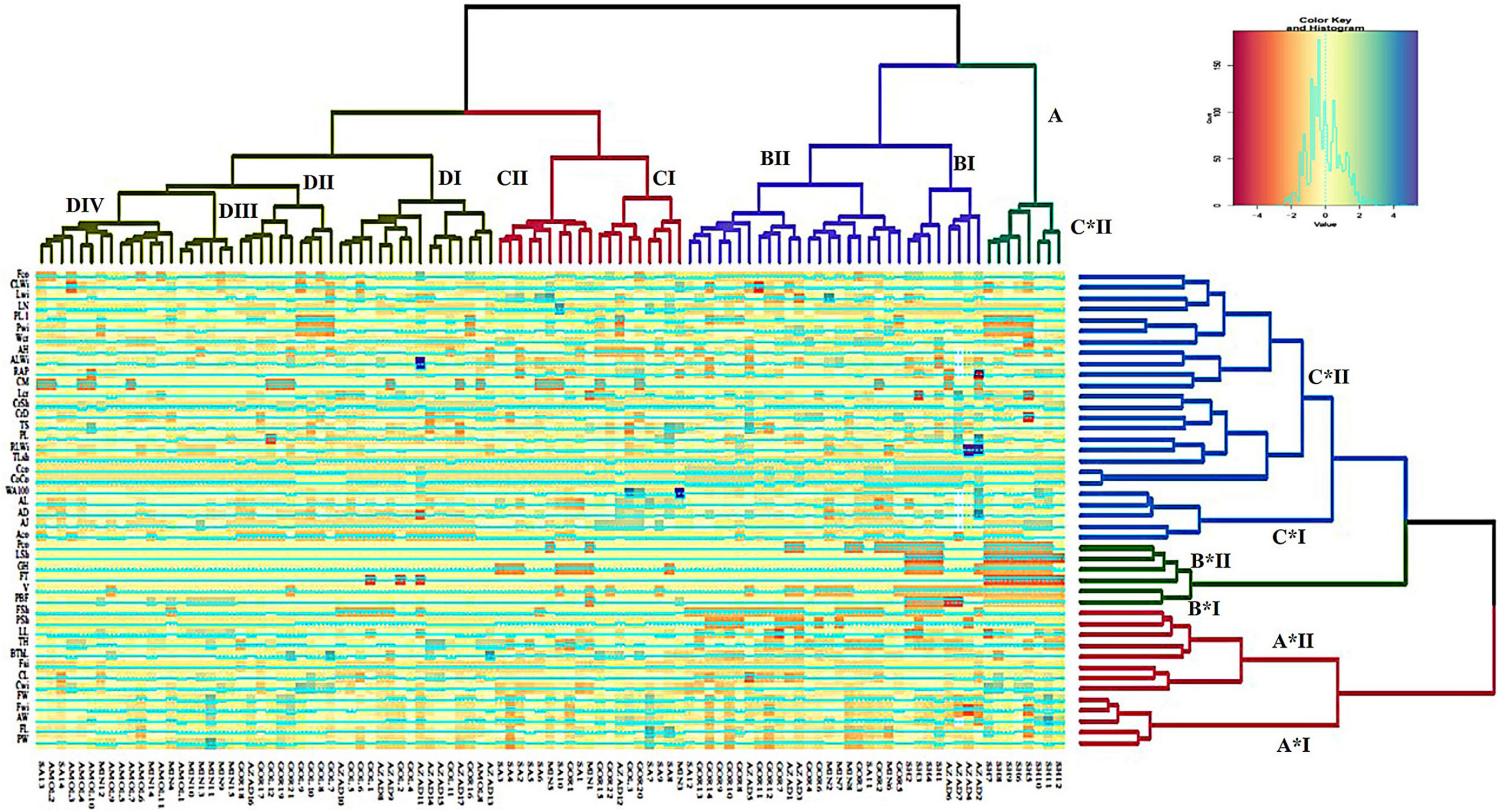
Cluster heatmap analysis of 103 wild pomegranate accessions based on the 45 studied traits.

The heatmap is a technique that visualizes the magnitude of a phenomenon in color in two dimensions. This analysis shows how genotypes are classified based on morphological traits. According to the results obtained from heatmap accessions clustering, the wild pomegranates were separated into four main groups, and considered traits in this study were divided into three main groups (Figure 8). The first group of accessions clustering contained eight accessions belonging to Shahroud (A). The second group (B) was divided into two subgroups so that the accessions related to Azadshahr and Shahroud were placed in the first subgroup (BI). In contrast, in the second subgroup (BII), a variety of accessions was observed, which were collected from Gorgan, Miankaleh, Sari, and Azadshahr. Also, the third group was subdivided into two subgroups so that the accessions collected from Miankaleh, Sari, Gorgan, Golestan forest, and Azadshar were placed in the first subgroup (CI) while the second subgroup (CII) included accessions collected from Miankaleh, Sari, and Gorgan. The fourth group was divided into four subgroups, so the first subgroup (DI) comprised the Sari, Azadshahr, Amol, Gorgan, and Golestan forest accessions. The accessions collected from Gorgan and Miankaleh were placed in the third subgroup (D III). The fourth subgroup comprised the accessions collected from Amol, Miankaleh, and Sari. The results of heatmap analysis (Figure 8) confirmed that the considered traits in this study were divided into three main groups. The first group (A*) was subdivided into two subgroups; the first subgroup (A*I) contained the peel weight, fruit length, fruit aril weight, fruit width, and fruit weight, whereas the second one (A*II) consisted of calyx width, calyx length, flower size, high thorn length, height, leaf length, petal shape, and flower shape. Based on the heatmap analysis, the second group was subdivided into two subgroups. The first subgroup (B*I) contained just two traits, percent of bell‐shaped flowers and vigor, and another one (B*II) consisted of flower type, growing habit, leaf shape, and petiole color which are identical to the A group in the accession cluster. Likewise, the third group is subdivided into two subgroups, in a way that the first subgroup (C*I) comprised aril color, aril juice, aril diameter, and aril length and weight of 100 arils, whereas the second subgroup (C*II) consists of corolla color, calyx color, tip leaf shape, fruit length/width ratio, petiole length, the thickness of peel, croon diameter, crown shape, fruit length of the crown, carob moth, aril percent, aril length/width ratio, aril hardiness, fruit width of the crown, petal width, petal length, leaf number per node, leaf width, calyx length/width ratio, and fruit over color (Figure 8). A total of 50% of the traits were classified as the third group and were found in the second subgroup (C*II), so these traits showed relatively high CVs, and thus, possessed high genetic variation. One of the effective factors for achieving maximum heterosis in breeding programs would be considering two completely inconsistent parents, one with the highest and the other with the lowest trait value (Ebrahimi & Alipour, 2020; El‐Sayed & Abbas, 2015). Grouping and characterizing germplasm provide valuable information for breeders and eliminates population resampling steps. One of the most common methods used by breeders in their breeding programs is selection along with generation testing. Access to heterosis and genetic recombination among the population is one of the success factors in the selection program. The possibility of heterosis in cross‐breeding programs with a rising genetic distance has been reported in many studies. Indeed, crosses occurring between genotypes at greater genetic distances can certainly lead to more recombination and heterosis (Subramanian & Subbaraman, 2010; Usharani et al., 2015). Grouping genotypes based on genetic distance can be a practical approach to breeding multiple traits.

## 4 CONCLUSION

In this study, the genetic variation of wild pomegranate in the north and northeast of Iran was evaluated by morphological traits. The results showed that the examined area has a relatively large genetic diversity. There are high variations in fruit cracking percent, crown shape, corolla color, calyx color, aril juiciness, aril color, length of thorn, the weight of arils in a fruit, weight of 100 arils, flower shape, susceptibility to carob moth, aril hardiness, fruit over color, and fruit weight in wild pomegranates. Our finding revealed superior accessions, including SH11, AZAD2, MIN3, MIN11, SA11, and GOL3, which can be applied to these as parents that certainly increase the chance of obtaining desirable genotypes in a breeding program. These accessions are of great value for most of the quantitative traits. The results provide valuable information about the identification of superior genotypes, genetic diversity, and the distribution of wild pomegranate genotypes. The results of this study provide information that can be effective in breeding programs for the development of superior genotypes as well as germplasm conservation programs and preventing the genetic drift in indigenous wild pomegranates in natural habitats.

## CONFLICT OF INTEREST

The authors have no conflict of interest.

## ETHICAL APPROVAL

This study does not involve any human or animal testing.

## Data Availability

The data that support the findings of this study are available from the corresponding author upon reasonable request.

## References

[fsn33135-bib-0001] Arlotta, C. , Toscano, V. , Genovese, C. , Calderaro, P. , Puglia, G. D. , & Raccuia, S. A. (2022). Nutraceutical content and genetic diversity share a common pattern in new pomegranate genotypes. Molecules, 27(2), 389. 35056703 10.3390/molecules27020389 PMC8779006

[fsn33135-bib-0002] Aziz, S. , Firdous, S. , Rahman, H. , Awan, S. I. , Michael, V. , & Meru, G. (2020). Genetic diversity among wild pomegranate (*Punica granatum*) in Azad Jammu and Kashmir region of Pakistan. Electronic Journal of Biotechnology, 46, 50–54.

[fsn33135-bib-0003] Chandra, R. , Babu, K. D. , Jadhav, V. T. , & Teixeira da Silva, J. (2010). Origin, history and domestication of pomegranate. Fruit, Vegetable and Cereal Science and Biotechnology, 2, 1–6.

[fsn33135-bib-0004] Ebrahimi, A. , & Alipour, H. (2020). Screening of wild superior apple genotypes in north and northeast of Iran using multivariate analysis. Euphytica, 216(9), 1–19.

[fsn33135-bib-0005] El‐Sayed, H. , & Abbas, I. K. (2015). Heterosis and combining ability in some bread wheat crosses. Egyptian Journal of Plant Breeding, 19(7), 2143–2154.

[fsn33135-bib-0006] Jalikop, S. , Kumar, P. , Rawal, R. , & Kumar, R. (2006). Breeding pomegranate for fruit attributes and resistance to bacterial blight. Indian Journal of Horticulture, 63(4), 352–358.

[fsn33135-bib-0007] Khadivi, A. , & Arab, M. (2021). Identification of the superior genotypes of pomegranate (*Punica granatum* L.) using morphological and fruit characters. Food Science & Nutrition, 9, 4570–4589. 10.1002/fsn3.2450 PMC8358378 34401105

[fsn33135-bib-0008] Khadivi, A. , Ayenehkar, D. , Kazemi, M. , & Khaleghi, A. (2018). Phenotypic and pomological characterization of a pomegranate (*Punica granatum* L.) germplasm collection and identification of the promising selections. Scientia Horticulturae, 238, 234–245.

[fsn33135-bib-0009] Khadivi, A. , Mirheidari, F. , Moradi, Y. , & Paryan, S. (2020). Morphological variability of wild pomegranate (*Punica granatum* L.) accessions from natural habitats in the northern parts of Iran. Scientia Horticulturae, 264, 109165.

[fsn33135-bib-0010] Khadivi‐Khub, A. (2015). Physiological and genetic factors influencing fruit cracking. Acta Physiologiae Plantarum, 37(1), 1718.

[fsn33135-bib-0011] Khan, M. A. , Khan, M. A. , Hussain, M. , & Mujtaba, G. (2014). Plant diversity and conservation status of Himalayan region Poonch Valley Azad Kashmir (Pakistan). Pakistan Journal of Pharmaceutical Sciences, 27(5), 1215–1239. 25176378

[fsn33135-bib-0012] Kher, R. (1999). A note on the physico‐chemical characters of the wild pomegranate (*Punica protopunica* L.). Annals of Biology, 15(2), 231–232.

[fsn33135-bib-0013] Maestre, J. , Melgarejo, P. , Tomas‐Barberan, F. , & Garcia‐Viguera, C. (2000). New food products derived from pomegranate. In Production, processing and marketing of pomegranate in the mediterranean region: Advances in research and technology (pp. 243–245). CIHEAM.

[fsn33135-bib-0014] Martinez, J. , Melgarejo, P. , Hernández, F. , Salazar, D. , & Martinez, R. (2006). Seed characterisation of five new pomegranate (*Punica granatum* L.) varieties. Scientia Horticulturae, 110(3), 241–246.

[fsn33135-bib-0015] Mirjalili, S. , & Poorazizi, E. (2015). Dispersion, biodiversity and genetic resources of pomegranate (*Punica granatum*) in Iran. Acta Horticulturae, 1089, 257–261.

[fsn33135-bib-0016] Mishra, G. , Sharma, G. , Taria, S. , & Negi, D. (2016). Study of different flower types in wild pomegranate germplasm in Western Himalayan zone. Indian Horticulture Journal, 6(3), 318–321.

[fsn33135-bib-0017] Nafees, M. , Jaskani, M. J. , Ahmed, S. , & Awan, F. S. (2015). Morpho‐molecular characterization and phylogenetic relationship in pomegranate germplasm of Pakistan. Pakistan Journal of Agricultural Sciences, 52(1), 97–106.

[fsn33135-bib-0018] Sarkhosh, A. , Zamani, Z. , Fatahi, R. , & Ranjbar, H. (2009). Evaluation of genetic diversity among Iranian soft‐seed pomegranate accessions by fruit characteristics and RAPD markers. Scientia Horticulturae, 121(3), 313–319.

[fsn33135-bib-0019] Shahsavari, S. , Noormohammadi, Z. , Sheidai, M. , Farahani, F. , & Vazifeshenas, M. R. (2022). A bioinformatic insight into the genetic diversity within pomegranate cultivars: From nuclear to chloroplast genes. Genetic Resources and Crop Evolution, 69(3), 1207–1217.

[fsn33135-bib-0020] Sharma, S. , & Sharma, V. (1990). Variation for chemical characters in some promising strains of wild pomegranate (*Punica granatum* L.). Euphytica, 49(2), 131–133.

[fsn33135-bib-0021] Sineh Sepehr, K. , Baradaran, B. , Mazandarani, M. , Khori, V. , & Shahneh, F. Z. (2012). Studies on the cytotoxic activities of *Punica granatum* L. var. spinosa (apple Punice) extract on prostate cell line by induction of apoptosis. ISRN Pharmaceutics, 2012, 547942. 10.5402/2012/547942 23320197 PMC3539436

[fsn33135-bib-0022] Singh, T. J. , & Gupta, T. (2018). Morphological and quality traits performance of the fruits of wild pomegranate (*Punica granatum* L.) in Himachal Pradesh. International Journal of Bio‐resource and Stress Management, 9(3), 341–344.

[fsn33135-bib-0023] Subramanian, A. , & Subbaraman, N. (2010). Hierarchical cluster analysis of genetic diversity in maize germplasm. Electronic Journal of Plant Breeding, 1(4), 431–436.

[fsn33135-bib-0024] Thakur, N. , Dhaygude, G. S. , & Gupta, A. (2011). Physico‐chemical characteristics of wild pomegranate fruits in different locations of Himachal Pradesh. International Journal of Farm Sciences, 1(2), 37–44.

[fsn33135-bib-0025] Thakur, N. , Thakur, A. , & Kumar, P. (2020). Effect of different drying modes on phenolics and antioxidant potential of different parts of wild pomegranate fruits. Scientia Horticulturae, 274, 109656.

[fsn33135-bib-0026] UPOV . (2012). Pomegranate. Guidelines for the conduct of tests for distinctness, uniformity and stability . https://www.upov.int/edocs/mdocs/upov/en/twf_43/tg_pgran.pdf

[fsn33135-bib-0027] Usharani, K. , Vindhiyavarman, P. , Balu, P. A. , & Boopathi, N. (2015). Heterosis studies for fibre quality traits in diallel crosses of upland cotton (*Gossypium hirsutum* L.). The Bioscan, 10(2), 793–799.

[fsn33135-bib-0028] Valero, C. , & Ruiz‐Altisent, M. (2000). Design guidelines for a quality assessment system of fresh fruits in fruit centers and hypermarkets. https://oa.upm.es/6398/

[fsn33135-bib-0029] Yilmaz, C. , Rezaei, M. , & Sarkhosh, A. (2021). Environmental requirements and site selection. In A. Sarkhosh , A. M. Yavari , & Z. Zamani (Eds.), The pomegranate: Botany, production and uses. (pp. 225–246). CABI.

[fsn33135-bib-0030] Zamani, Z. , Adabi, M. , & Khadivi‐Khub, A. (2013). Comparative analysis of genetic structure and variability in wild and cultivated pomegranates as revealed by morphological variables and molecular markers. Plant Systematics and Evolution, 299(10), 1967–1980.

[fsn33135-bib-0031] Zarei, F. , Karimi, H. R. , Mirdehghan, S. H. , & Mohammadi Mirik, A. A. (2018). Genetic diversity among Iranian pomegranate (*Punica granatum* L.) varieties by morphological markers. *Iranian* . Journal of Horticultural Science, 48(4), 811–821. 10.22059/ijhs.2018.202565.963

[fsn33135-bib-0032] Zuriaga, E. , Pintová, J. , Bartual, J. , & Badenes, M. L. (2022). Characterization of the Spanish pomegranate germplasm collection maintained at the agricultural Experiment Station of Elche to identify promising breeding materials. Plants, 11(9), 1257. 10.3390/plants11091257 35567258 PMC9101082

